# Endoscopic versus open surgery for insertional achilles tendinopathy: A systematic review and meta‐analysis of comparative outcomes

**DOI:** 10.1002/jeo2.70374

**Published:** 2025-07-24

**Authors:** Po‐Yuan Chen, I‐Shiang Tzeng, Kai‐Chiang Yang, Chen‐Chie Wang

**Affiliations:** ^1^ Department of Orthopedic Surgery, Taipei Tzu Chi Hospital Buddhist Tzu Chi Medical Foundation New Taipei City Taiwan; ^2^ Department of Research, Taipei Tzu Chi Hospital Buddhist Tzu Chi Medical Foundation New Taipei City Taiwan; ^3^ School of Dental Technology, College of Oral Medicine Taipei Medical University Taipei Taiwan; ^4^ Department of Orthopedics, School of Medicine Tzu Chi University Hualien Taiwan

**Keywords:** endoscopic, Haglund's deformity, Haglund's syndrome, insertional Achilles tendinopathy, retrocalcaneal bursitis

## Abstract

**Purpose:**

Insertional Achilles tendinopathy (IAT) causes chronic hindfoot pain and functional impairment. Although conservative treatment remains the first‐line management approach, surgery is often necessary when nonoperative measures fail. Both open and endoscopic techniques are commonly used, but their comparative efficacy remains debated. Accordingly, this meta‐analysis compared surgical outcomes, complications and recovery between open and endoscopic techniques; it also conducted a subgroup analysis to assess return to sports in highly active individuals.

**Methods:**

A systematic literature search was conducted in PubMed, the Cochrane Library, Scopus, ScienceDirect, Web of Science and Embase (2003–2024). Studies were included if they reported outcomes for open or endoscopic IAT surgery with ≥20 patients and ≥6 months of follow‐up. Outcomes included the American Orthopaedic Foot and Ankle Society (AOFAS) scores, time to return to sports, complication rates and additional functional outcome measures.

**Results:**

Thirty‐nine studies (1559 patients, 1625 procedures) were included. Mean AOFAS scores improved from 56.07 to 89.17 (*p* < 0.001), with no significant difference between surgical techniques (*p* = 0.18). However, endoscopic surgery was associated with a lower complication rate and faster recovery, enabling earlier return to daily activities (6.75 ± 2.25 vs. 22.45 ± 4.74 weeks, *p* < 0.001) and sports (12.63 ± 2.2 vs. 22.13 ± 7.42 weeks, *p* < 0.001). Among highly active individuals, endoscopic surgery facilitated return to sports within 12–18 months, whereas open surgery required 20–30 months.

**Conclusions:**

Endoscopic surgery demonstrates a low complication rate and expedited recovery, making it a preferable option for patients requiring an early return to activity. More high‐quality studies, such as randomized controlled trials and standardized protocols, are needed to improve surgical decisions and treatment strategies for IAT.

**Level of Evidence:**

Level IV.

**Abbreviations**
AOFAS: American Orthopaedic Foot and Ankle Society
AT: Achilles tendon
BMI: body mass index
IAT: insertional Achilles tendinopathy
INPLASY: International Platform of Registered Systematic Reviews
MINORS: Methodological Index for Non‐Randomized Studies
post‐OP: post‐operative
pre‐OP: preoperative
RCT: randomized controlled trial
RevMan: Review Manager
ROM: range of motion
SD: standard deviation
VAS: visual analogue scale
VISA‐A: Victorian Institute of Sport Assessment‐Achilles

## INTRODUCTION

Insertional Achilles tendinopathy (IAT) is a chronic condition characterized by persistent hindfoot pain, painful range of motion (ROM), and localized swelling. It is often associated with repeated stress from athletic activities, rigid heel counters, and pes cavovarus deformity. Additionally, IAT commonly coexists with retrocalcaneal bursitis and Haglund's deformity [49]—an abnormal bony enlargement of the posterior calcaneal tuberosity that exacerbates Achilles tendon (AT) chafing, leading to tendon degeneration and an increased risk of tearing [44, 53, 60, 61].

This condition predominantly affects individuals aged 20–60 years, and its prevalence is particularly high among athletes involved in running and jumping sports, which account for 5%–18% of all running‐related injuries [54, 65]. Maffulli et al. [38] defined Achilles tendinopathy as a clinical syndrome characterized by pain, swelling, and functional impairment; these symptoms are consistent with the histological features of tendinosis, a degenerative process marked by collagen disorganization.

The diagnosis of IAT is based on a combination of clinical assessment and imaging modalities. Lateral foot X‐rays often reveal Haglund's deformity, intratendinous calcifications, and an increased Fowler–Philip angle (>75°) [49], indicating chronic stress [34, 53]. Ultrasonography typically demonstrates tendon thickening (>8 mm), hypoechoic degeneration, and increased vascularity. Moreover, magnetic resonance imaging provides a detailed visualization of T2 hyperintensity at the insertion site, tendon oedema and bone marrow involvement, facilitating differential diagnosis and treatment planning [44, 53, 60, 61].

Conservative treatment remains the first‐line approach for managing IAT; such treatment focuses on pain reduction, inflammation control, and gradual tendon loading to restore function. Eccentric exercises, particularly the Alfredson protocol [3], are widely recommended. Effective load management is crucial and requires a structured return‐to‐activity strategy for preventing symptom exacerbation. Extracorporeal shockwave therapy has demonstrated promising results, with studies reporting significant pain reduction and functional improvement, particularly when combined with exercise therapy [44, 53]. Nonsteroidal anti‐inflammatory drugs and corticosteroid injections may provide temporary symptom relief. However, they do not address the underlying tendon pathology and may increase the risk of further degeneration. Regenerative treatments, including platelet‐rich plasma and high‐volume injections, have been explored, although current evidence remains inconclusive. Additionally, heel lifts and orthotic devices can reduce mechanical stress at the AT insertion, leading to symptomatic improvement in some cases [44, 53, 65]. When conservative management fails to yield satisfactory outcomes after a sufficient trial period—typically 6 months—surgical intervention is recommended.

Several surgical techniques have been proposed for IAT treatment, including open debridement, arthroscopic surgery, Zadek osteotomy [68], ultrasound‐guided osteotomy and tendon scraping [10, 44, 45]. Among these, open and endoscopic techniques are the most widely applied and extensively studied [2]. The open technique provides superior visualization of the lesion site and typically involves the excision of degenerated tendon tissue, resection of Haglund's deformity or wedge osteotomy. However, this technique is associated with a relatively high risk of post‐operative complications, including wound infection, hindfoot stiffness, delayed wound healing, surgical site paraesthesia, and even AT rupture [44, 53, 60].

The endoscopic technique is a minimally invasive approach characterized by smaller incisions, reduced recovery time and enhanced healing outcomes. However, it necessitates a steeper learning curve for surgeons because of its technical complexity. The endoscopic technique mitigates several risks associated with the open technique; nevertheless, Alessio‐Mazzola et al. [2] reported complications such as infection, delayed wound healing, paraesthesia and AT rupture [44, 45, 60].

The present study provides a comparative analysis of long‐term outcomes, complication rates, and recovery times between the open and endoscopic techniques by using up‐to‐date data. The findings provide insights to inform clinical decision‐making.

## METHODS

**Figure 1:**
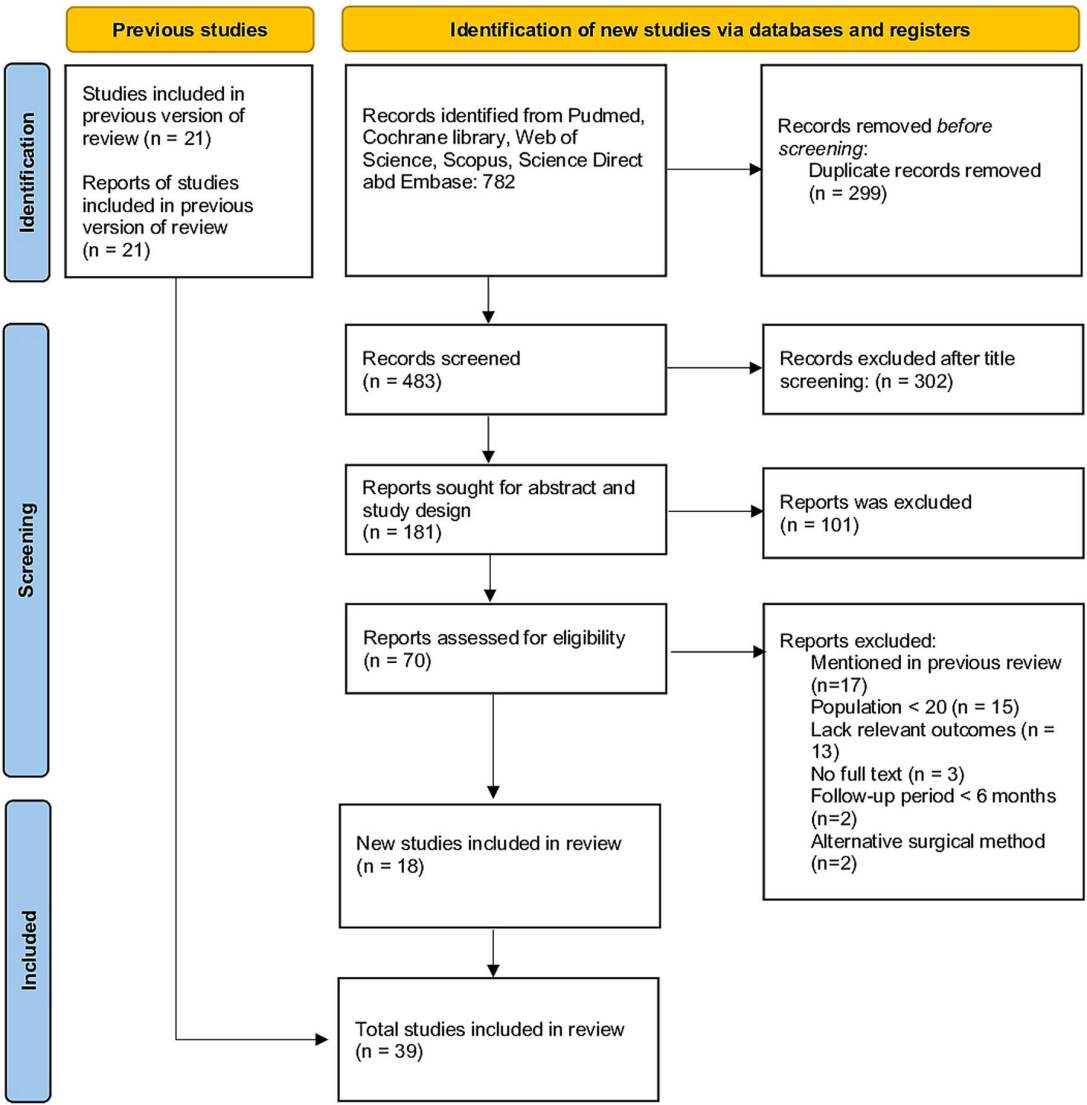
Prisma flow diagram for study selection.

This systematic review and meta‐analysis compared endoscopic and open surgical procedures for IAT treatment. The study was conducted following the Preferred Reporting Items for Systematic Reviews and Meta‐Analyses guidelines [43] (Figure 1 and Table S1). This study has been registered on the INPLASY (registration number: INPLASY20250122).

### Search strategy and selection criteria

Two investigators, A and B, independently searched PubMed, Cochrane Library, Scopus, Science Direct, Web of Science and Embase for studies published between 1 January 2003 and 1 December 2024. The following combinations of search terms were used: (‘Haglund’ OR ‘retrocalcaneal bursitis’ or ‘insertional Achilles tendinopathy’) AND (‘reinsertion’ OR ‘reattachment’ OR ‘debridement’ OR ‘retrocalcaneal decompression’) and (‘Haglund’ OR ‘retrocalcaneal bursitis’ or ‘insertional Achilles tendinopathy’) AND (‘Endoscopy’ OR ‘Arthroscopy’ OR ‘Minimally invasive procedure’) (Table S2).

Two investigators, A and B, independently screened the titles, abstracts, patient sample sizes and full‐text articles to identify eligible studies. Studies were included if they reported clinical outcomes of either open or endoscopic surgical procedures for IAT, included ≥20 patients, and had a follow‐up period of ≥6 months. Duplicate publications and studies not published in English were excluded.

Case reports, animal studies, cadaveric studies, commentaries, technical studies and clinical studies lacking methodological details or sufficient quantitative or qualitative data were excluded from the analysis. Additionally, studies with fewer than 20 patients, those published before 1 January 2003, or those with a follow‐up period of <6 months were excluded. Studies involving gastrocnemius recession, Zadek osteotomy or flexor hallucis longus tendon transfer were also excluded.

To ensure comprehensive coverage, we applied a snowballing technique. This technique involved manual screening relevant articles, the reference lists of included studies, and articles citing these studies to identify additional eligible publications.

The primary outcome of this study was the mean American Orthopaedic Foot and Ankle Society (AOFAS) score [26], a clinician‐reported measure of pain, function, and alignment in patients with foot and ankle conditions. Secondary outcomes included the mean time to return to daily activities or sports, complication rates, Victorian Institute of Sport Assessment‐Achilles (VISA‐A) score [22]—a patient‐reported measure of Achilles tendinopathy severity based on pain, function and activity levels—and visual analogue scale (VAS) score [30], a measure of pain intensity along a continuous spectrum.

### Data extraction

Two investigators, A and B, independently extracted data from the included studies. The extracted data included the following: first author name, publication year, study country, study design, surgical intervention type, patient count, procedural details, procedure laterality, sex distribution, mean age, follow‐up duration, diagnostic methods, intervention details, rehabilitation protocol and mean time to return to daily activities and sports.

### Quality assessment and risk of bias

Two reviewers, A and B, independently assessed the quality of the included articles. In cases where consensus could not be reached, a third reviewer, C, conducted an independent evaluation to resolve discrepancies.

Study quality was assessed using the Grading of Recommendations, Assessment, Development and Evaluation system [19], and the level of evidence was determined according to the Oxford Centre for Evidence‐Based Medicine 2011 criteria [50]. Risk of bias was assessed using the Methodological Index for Non‐Randomized Studies (MINORS) [62], which assigns a maximum score of 24 for comparative studies and 16 for single‐group studies. Comparative studies were categorized as having a high risk of bias if their total MINORS score was ≤16 and as having a low risk of bias if their total MINORS score was >16. Similarly, single‐group studies with a total MINORS score of ≤12 were deemed to have a high risk of bias, whereas those scoring >12 were considered to have a low risk of bias.

Publication bias was assessed through visual inspection of a funnel plot for the primary outcome. A symmetrical distribution of studies around the pooled effect estimate was interpreted as indicating a low risk of small‐study effects or publication bias. The analysis was performed using Review Manager (RevMan) version 5.4 (Cochrane Collaboration).

### Statistical analysis and meta‐analysis

All continuous variables, including AOFAS scores, additional functional assessments, mean time to return to daily activities and sports, and complication rates, are reported as means with standard deviations (SDs) or ranges. Continuous outcomes were pooled using the inverse variance weighting method and are presented as mean differences with 95% confidence intervals. All analyses were conducted using random‐effects models.

We compared the changes in AOFAS scores from baseline to the final follow‐up for each surgical procedure to evaluate functional improvement. Complications, including infection, paraesthesia, hypertrophic scars, residual pain and surgical failures (e.g., AT rupture, recurrence and the need for secondary surgery), were recorded and analyzed to compare complication rates between the open and endoscopic techniques. The mean time to return to daily activities and sports was recorded and analyzed; it is reported as mean ± SD.

To further investigate recovery patterns, we conducted a subgroup analysis by selecting studies that included highly active populations, such as athletes or young active individuals. This subgroup was compared with the general population in terms of recovery speed, functional outcomes and complication rates.

All statistical analyses were conducted using Review Manager (RevMan) 5.4 software (Cochrane Collaboration). Changes in AOFAS scores from preoperative (pre‐OP) to post‐operative (post‐OP) time points were analyzed. Study heterogeneity was assessed using the *I* ^2^ statistic, with *I* ^2^ values of <40% indicating low heterogeneity, values of 40%–75% indicating moderate heterogeneity, and values of >75% indicating substantial heterogeneity. A *p* value of <0.05 was considered statistically significant.

## RESULTS

### Search results and study inclusion

The search strategy and study selection process are presented in Figure 1. The previous version of this review included 35 studies, of which 21 were published after 2003 and met our inclusion criteria [1, 4, 5, 6, 8, 9, 14, 20, 23, 24, 25, 27, 32, 33, 41, 47, 52, 58, 66, 67]. In this updated review, a total of 782 studies were initially identified. We removed 299 duplicate records and excluded 413 studies after screening the titles, abstracts and study types on the basis of the predefined eligibility criteria. Among the remaining 70 studies, 17 overlapped with the previous review, 15 had a sample size of <20 patients, 13 lacked relevant outcome measures, 3 had no full text available, 2 failed to meet the required minimum follow‐up period and 2 employed alternative surgical methods that did not align with the study criteria. As a result, a total of 18 additional studies were included in our study.

After incorporating 21 studies from the previous review and 18 additional studies, 39 studies were included in this systematic review and meta‐analysis. Notably, no randomized controlled trials (RCTs) were identified. All included studies were observational, comprising 11 prospective studies [6, 27, 29, 32, 35, 36, 37, 39, 40, 42, 64] and 28 retrospective studies [1, 4, 5, 6, 7, 8, 9, 13, 14, 15, 16, 17, 18, 20, 23, 24, 25, 31, 33, 41, 46, 47, 52, 55, 58, 59, 66, 67].

### Characteristics of the included studies

The 39 studies included in the analysis involved 1559 patients who underwent 1625 surgical procedures. Among the 39 studies, 5 directly compared open and endoscopic techniques [12, 32, 40, 55, 64], whereas 23 exclusively focused on open surgery [4, 5, 7, 8, 9, 13, 15, 16, 17, 18, 20, 24, 29, 31, 33, 35, 36, 37, 39, 41, 47, 59, 67] and 11 solely focused on endoscopic surgery [1, 4, 6, 14, 23, 25, 27, 42, 46, 52, 58, 66]. The studies were conducted across multiple regions: 13 in Europe [7, 12, 13, 17, 20, 23, 35, 36, 37, 39, 41, 58, 64], 15 in Asia [6, 15, 18, 24, 25, 27, 29, 31, 33, 42, 46, 47, 55, 66, 67], 6 in the Americas [5, 8, 16, 32, 52, 59], 4 in Egypt [1, 4, 14, 40] and 1 in Australia [9].

The overall mean age of the patients in the included studies was 46.18 ± 8.21 years. The mean follow‐up period was 34.04 ± 15.13 months.

The endoscopic group contained 611 patients and 637 ft, whereas the open surgical group comprised 948 patients and 988 ft. The open surgical group had a mean age of 46.77 ± 10.25 years and a mean follow‐up period of 32.51 ± 11.81 months. By contrast, the endoscopic surgical group had a mean age of 42.78 ± 9.16 years and a mean follow‐up period of 36.29 ± 14.57 months.

Table 1 presents the baseline characteristics of the patients in each study, and Table 2 presents a summary of the treatment characteristics among the included studies.

**Table 1:** Characteristics of the included studies.

Source	Country	Year	Number of patients	Number of procedures	Sex	Type of intervention	Side	Mean age	Follow‐up (range)	Study design	Level of evidence	Grade
Thiounn [63]	France	2024	85	85	‐	Endoscopic vs. Open	L: 39	49.16 ± 12.53	≥6 mo	Prospective cohort study	II	Moderate
							R: 46					
Lugani [34]	Italy	2023	42	47	M: 22	Open	‐	53 (23–78)	≥12 mo	Prospective case series	IV	Low
					F: 20							
Nakajima [45]	Japan	2022	44	44	M: 31	Endoscopic	‐	55.7 ± 11.0 (35‐77)	2.8 + 0.7 yr	Retrospective case series	IV	Very low
					F: 13							
Scott [58]	USA	2022	38	40	M: 8	Open	L: 20	54.52 (32–76)	32.5 mo (13–53)	Retrospective cohort study	IV	Very low
					F: 30		R: 20					
Maffulli [36]	Italy	2022	33	33	M: 24	Open	L: 19	43.9 ± 10.6 yr	≥24 mo	Prospective case series	IV	Low
					F: 9		R: 14					
Baumbach [7]	German	2022	88	88	M: 58	Open	L: 35	50 ± 12 yr (47–52)	3.8 ± 1.9 yr (3.4–4.3)	Retrospective case series	III	Low
					F: 30		R: 53					
Lee [30]	Korea	2021	20	20	M: 19	Open	‐	19.9 ± 4.92 yr	31.3 ± 8.95 mo	Retrospective case series	IV	Very low
					F: 1							
Greiner [16]	Austria	2021	42	42	M: 16	Open	‐	56.8 ± 10.2 yr (27–73)	32.8 ± 14.2 mo (18–52)	Retrospective case series	IV	Very low
					F: 26							
Cusumano [11]	Italy	2021	54	54	M: 31	Endoscopic vs. Open		49 ± 9 yr	53.8 ± 24 mo	Retrospective cohort study	IV	Very low
					F: 23							
Pi [54]	China	2021	47	47	M: 36	Endoscopic vs Open	‐	37 ± 12 yr	38 ± 18 mo	Retrospective cohort study	IV	Very low
					F: 11							
Mishra [41]	India	2020	23	32	M: 14	Endoscopic	L: 17	‐	12 mo	Prospective case series	IV	Very low
					F: 9		R: 15	(20–70 yr)				
Ge [14]	China	2020	32	32	M: 26	Open	L: 14	36 ± 14.1 yr	71.8 ± 22.4 mo	Retrospective case series	IV	Low
					F: 6		R: 18					
Yasin [17]	Turkey	2020	27	27	M: 13	Open	L: 13	47 ± 8 yr	31 ± 5 mo	Retrospective case series	IV	Very low
					F: 14		R: 14					
Allam [4]	Egypt	2019	21	21	M: 13	Open	‐	42 yr (32–56)	8.6 ± 1.2 mo (6‐12)	Retrospective case series	IV	Very low
					F: 8							
Mir [40]	Italy	2018	25	29	M: 9	Open	L: 7	38.7 yr (25–60)	13.6 mo (12–16)	Retrospective case series	IV	Very low
					F: 16		R: 10					
Hardy [19]	France	2018	46	46	M: 40	Open	L: 19	44.1 ± 11.4 yr	33 ± 13.5 mo	Retrospective case series	IV	Low
					F: 6		R: 27					
Barsan [6]	India	2018	22	27	‐	Endoscopic	‐	‐	12 mo	Prospective case series	III	Low
Xia [66]	Singapore	2017	22	22	M: 10	Open	L: 11	59 ± 7.3 yr	15.1 mo (12–26)	Retrospective case series	IV	Low
					F: 12		R: 11					
Mansour [39]	Egypt	2017	26	34	M: 9	Endoscopic vs Open	L: 15	31 yr (24–56)	20 mo (16–28)	Prospective cohort study	II	Low
					F: 17		R: 19					
Ewais [13]	Egypt	2017	22	26	M: 6	Endoscopic	‐	24.3 yr (18–35)	12 mo (8–15)	Retrospective case series	IV	Very low
					F: 16							
Aldahshan [1]	Egypt	2017	50	50	M: 26	Endoscopic	L: 26	43.1 yr (22–59)	2.2 ± 1.1 yr (0.5–4)	Retrospective case series	IV	Very low
					F: 24		R: 24					
Jiang [23]	China	2016	32	32	M: 11	Open	L: 15	51.4 yr (21–68)	3.5 yr (24–60 mo)	Retrospective cohort study	III	Low
					F: 21		R: 17					
Hong [28]	Singapore	2016	22	22	M: 12	Open	L: 14	55.3 ± 9.2 yr	21.5 ± 8.2 mo (7–35)	Prospective case series	IV	Very low
					F: 10		R: 8					
Jerosch [22]	Germany	2015	164	164	‐	Endoscopic	‐	16‐64 yr	46.3 mo (8–120)	Retrospective case series	IV	Low
Natarajan [46]	India	2015	40	46	M: 12	Open	‐	44 yr (38–50)	13 mo (12–15)	Retrospective case series	IV	Very low
					F: 28							
Ettinger [12]	Germany	2015	40	40	M: 19	Open	L: 19	52.3 ± 10.5 yr	15.6 ± 3.7 mo (12–27)	Retrospective case series	IV	Low
					F: 21		R: 21					
Lin [32]	Singapore	2014	44	44	M: 15	Open	‐	53 yr	6.1 mo	Retrospective case series	IV	Very low
					F: 29							
Greenhagen [15]	USA	2013	30	30	M: 10	Open	L: 12	49.13 ± 9.19 yr	28.93 ± 16.99 mo	Retrospective case series	IV	Low
					F: 20		R: 18					
Kaynak [24]	Turkey	2013	28	30	M: 18	Endoscopic	‐	37 yr (19–64)	58.4 mo (24–75)	Retrospective case series	IV	Low
					F: 10							
Wu [65]	China	2012	23	25	M: 6	Endoscopic	‐	27.7 yr (17–41)	41 mo (30–59)	Retrospective case series	IV	Low
					F: 17							
Kondreddi [26]	India	2012	23	25	M: 9	Endoscopic	‐	51.44 ± 7.92 yr (38–66)	13.4 ± 6.67 mo (6–30)	Prospective case series	III	Very low
					F: 14							
Maffulli [35]	Italy	2011	30	30	M: 21	Open	‐	48.9 yr (35–64)	39 mo (37–73)	Prospective case series	IV	Low
					F: 9							
Anderson [5]	USA	2008	62	66	M: 22	Open	‐	50.5 yr (19–82)	34.6 mo (12–109)	Retrospective cohort study	III	Low
					F: 40							
Ortmann [51]	USA	2007	28	30	M: 14	Endoscopic	L: 15	51 yr (22–75)	35 mo (3–62)	Retrospective case series	IV	Very low
					F: 16		R: 15					
Scholten [57]	Netherlands	2006	36	39	M: 20	Endoscopic	L: 18	35 yr (15–50)	4.5 yr (2–7.5)	Retrospective case series	IV	Low
					F: 16		R: 21					
Brunner [8]	USA	2005	36	38	M: 9	Open	‐	49 yr (19–81)	51 mo (15–109)	Retrospective case series	IV	Very low
					F: 27							
Maffulli [38]	Italy	2004	21	21	M: 15	Open	‐	46.9 ± 6.4 yr	20.2 mo (14–45)	Prospective case series	IV	Low
					F: 6							
Leitze [31]	USA	2003	44	50	M: 14	Endoscopic vs. Open	‐	50 yr (15‐79)	28.4 mo (6–52)	Prospective cohort study	II	Moderate
					F: 30							
Calder [9]	Australia	2003	45	52	M: 33	Open	‐	48 yr (18–66)	8 mo (6–22)	Retrospective case series	IV	Very low
					F: 12							

Abbreviations: F, female; Grade, Grading of Recommendations, Assessment, Development and Evaluations; L, left; M, male; Mo, month(s); R, right; yr, year(s).

**Table 2:** Clinical outcome of the included studies.

				Intervention					AOFAS			Mean time to return		
Source	Type of intervention	Number of procedures	Diagnosis method	Position	Anaesthesia	Approach	Procedure	Rehabilitation protocol	Pre	Post	Other measure	Daily life	Sport	Complication
Thiounn et al. [63]	E	51	X‐ray, MRI	‐	‐	‐	‐	‐	‐	‐	VISA‐A: Pre‐OP: 41.36 ± 18.02 (10–78)	‐	‐	1 case of symptomatic pre‐Achilles bursitis
											Post‐OP: 76.11 ± 19.91 (40––100)			
											EFAS life: pre‐OP: 9.83 ± 4.86			
											Post‐OP: 18.37 ± 5.75			
											EFAS sports: pre‐OP: 4.16 ± 4.4			
											Post‐OP: 10.22 ± 6.29			
	O	34		‐	‐	‐	‐	‐	‐	‐	VISA‐A: Pre‐OP: 33.81 ± 20.39 (8–77)	‐	‐	‐
											Post‐OP: 76.5 ± 20.93 (13–100)			
											EFAS life: Pre‐OP: 8.22 ± 4.53			
											Post‐OP: 17.86 ± 5.46			
											EFAS sports: Pre‐OP: 2.53 ± 2.97			
											Post‐OP: 9.14 ± 6.84			
Lugani et al. [34]	O	47	X‐ray	Prone	‐	Median/lateral	AT debridement, AT detachment, Bursectomy, calcaneal osteotomy, AT reattachment and repair, suture anchors	NWB in knee brace for 2w, then Gradual progressive PWB after suture removal	46.5 (30–91)	95 (85–100)	VISA‐A:	‐	‐	1 case of a small
											Pre‐OP: 47.8 (2–90)			Recurrent
											Post‐OP: 90 (77–100)			Intratendinous
Nakajima et al. [45]	E	44	3D‐CT, MRI	Prone	‐	‐	2.3‐mm 30° arthroscopy, AT debridement, 3.0‐mm hooded abrasion bur, 3.5‐mm cutter, Calcaneoplasty, bursectomy, fluoroscopic guidance	FWB with the splint, passive ROM exercises at 3 w, splint removed at 3–4w, return to jogging at 2 m, return to unrestricted sport at 3 m	‐	‐	VISA‐A (22 athletes)	‐	4.5 (2–12) m	2 cases of oversensitive scar
											Pre‐OP: 40.5 (12–65)			
											Post‐OP:95.0 (64–100)			
											VAS: Pre‐OP: 6.45 (2.5–10)			
											Post‐OP: 0.65 (0–4.6)			
											JSSF: Pre‐OP: 67.0 (22–92)			
											Post‐OP: 100 (78–100)			
Scoott et al. [58]	O (knotted)	24	‐	Prone	‐	Midline incision/AT split	Central tendon splitting, Bursectomy, Calcaneoplasty, double‐row repair (knotted/knotless), rongeur	NWB for 2w in the splint, then WB as tolerated in a long boot and begin gentle ankle ROM exercise, then low impact exercises in regular shoes at 6w, then high impact exercises at 6 m	‐	‐	VAS: Pre‐OP: 6.33	‐	‐	1 case of wound dehiscence,
	O(knotless)	16									Post‐OP: 1.96			1 case of deep abscess
									‐	‐	VAS: Pre‐OP: 5.75	‐	‐	Nil
											Post‐OP: 1.18			
Maffulli et al. [36]	O	33	X‐ray, MRI, ultrasound	Prone	Spinal/general	Cincinnati incision	AT debridement, Bursectomy, Calcaneal resection	WB as tolerated in plaster shoes with walker, then cast removal at 2w and stationary cycling/weight‐bearing were recommended, then rehabilitation from 6th to 12th w, then start jogging at 6 m	‐	‐	VISA‐A: pre‐OP: 39.7 ± 23	‐	‐	Nil
											Post‐OP: 74.5 ± 29.1			
											EQ.5D (post‐op): activity: 1.3 ± 0.6			
											Anxiety‐depression: 1.2 ± 0.4			
											Mobility: 1.2 ± 0.4			
											Pain/discomfort: 1.5 ± 0.5			
											Self‐care: 1.0 ± 0.0			
Baumbach et al. [7]	O	88	X‐ray, MRI	Prone	‐	Midline incision/AT split	AT detachment, AT debridement, AT reattachment, suture anchors	Neutral position with walker for 6–8w, then 10 kg PWB for 2w, then FWB	‐	‐	VISA‐A: Post‐OP: 81 ± 22 (95% CI: 77–86)	‐	‐	32 cases with complications (infection, major)
											SF‐12 PCS: 54 ± 7 (95% CI: 52–55)			
											SF‐12 MCS: 52 ± 9 (95% CI: 50–54).			
											F‐P angle not satisfied in 5%, intermediate in 18%, and very satisfied in 77%.			
Lee et al. [30]	O	20	X‐ray	Semilateral position	‐	lateral	AT debridement, retrocalcaneal decompression, bursectomy, calcaneal resection, drain insertion	Drain removal 1 day after surgery and NWB with short leg splint for 3w, then ROM exercise at 2w, then ROM and Achilles tendon strengthening in walking boot orthosis at 3w then eccentric stretching at 4w	65.1 ± 8.46	88.4 ± 7.08	Time of RTC, months: RTP group: 5.0 ± 1.41	‐	7.45 m (60% back to sport)	Nil
											Non‐RTP group: 11.1 ± 3.04			
											9 excellent, 6 good, 3 fair, 2 poor			
Greiner et al. [16]	O	42	X‐ray, MRI	Prone	‐	Midline incision/AT split	Central tendon splitting, AT debridement, calcaneal osteotomy, AT reattachment, 4 bone anchors (Double‐Row Refixation)	Cast for 6w including NWB in an equinus position for 2w, partial WB for 2w and FWB in a plantigrade position for 2w, then FWB with walker orthosis for 2w	51.0 ± 12.5	91.3 ± 14.3	FFI‐P: Pre‐OP: 54.8 ± 15.5	‐	‐	1 case of hypertrophic scar tissue
											Post‐OP: 8.1 ± 15.8			2 cases of superficial wound infection
											VAS: Pre‐OP: 8.9 ± 1.0			
											Post‐OP: 1.5 ± 2.5			
											FAOS (pain) pre‐OP: 36.5 ± 15.3			
											Post‐OP: 87.9 ± 18.1			
Cusumano et al. [11]	E	26	X‐ray	Prone	Regional	Two‐portal	Bursectomy, calcaneoplasty	Gradually weight‐bear at tolerate after surgery	66.69 ± 7.19	93.69 ± 10.04	FFI‐P: Pre‐OP: 55.85 ± 12.28	‐	‐	2 cases of wound infection
											Post‐OP: 9.64 ± 16.51			
											VAS: Pre‐OP: 7.57 ± 1.27			
											Post‐OP: 1.30 ± 1.93			
	O	28		Prone	spinal	Lateral	AT central split, bursectomy, calcaneal resection, AT reattachment and repair	NWB in cast for 2w, then cast removal and NBW active ROM with articulated splint, then athletic activity resumed at 12w	65.67 ± 10.09	91.78 ± 9.67	FFI‐P: Pre‐OP:52.19 ± 13.33	‐	‐	1 case of AT rupture
											Post‐OP: 8.38 ± 17.08			
											VAS: Pre‐OP: 6.32 ± 1.36			
											Post‐OP: 1.21 ± 1.93			
Pi et al. [54]	E	27	MRI	Prone	‐	Two‐portal	Bursectomy, Calcaneoplasty, AT repair	Plastic brace in the plantarflexion position for 2w, then WB walking in walking boot for 2w, then walk without restrictions and gradually return to their preoperative activities	‐	92.1 ± 8.0	VAS: Post‐OP: 3.7 ± 4.7	‐	‐	Nil
											Tengner score: 3.9 ± 1.9			
											AAS: 5.0 ± 2.5			
											SF‐36 (physical): 87.3 ± 13.2			
											SF‐36 (mental): 91.3 ± 14.0			
											C‐L angles			
											F‐P angles			
	O	20		Prone	‐	Lateral	AT Debridement, bursectomy, calcaneoplasty		‐	96.1 ± 5.1	VAS: Post‐OP: 2.1 ± 2.7	‐	‐	2 cases of transient paraesthesia at surgical site
											Tengner score: 3.2 ± 1.2			
											AAS: 4.1 ± 1.6			
											SF‐36 (physical): 86.5 ± 9.9			
											SF‐36 (mental): 96.8 ± 5.9			
											C‐L angles			
											F‐P angles			
Mishra et al. [41]	E	32	X‐ray, Ultrasound	Prone	Spinal	Two‐portal	4 mm arthroscopy, AT debridement, bursectomy, calcaneal resection, endoscopic shaver	NWB for 2w, then PWB as tolerate with brace for 2–3w, then normal walk at 4w	66.96 ± 6.45	95.20 ± 3.14	Excellent: 21	‐	‐	2 cases of paraesthesia in sural nerve distribution
											Good: 2			
Ge et al. [14]	O	32	X‐ray	Lateral	Spinal	Lateral	Calcaneal resection	NWB and active ROM at first 3w, then walk with a cane, then FWB at 6w	50.7 ± 5.1	93.4 ± 6.1	VISA‐A: Pre‐OP: 35.7 ± 7.1	‐	‐	1 case of S. aureus infection at the incision site
											Post‐OP: 94.3 ± 5.0			
											Fowler–Philip angle			
											Bohler's angle			
											Calcaneal pitch angle			
Yasin et al. [17]	O	27	X‐ray	Prone	Regional	Midline incision/AT split	AT central split, bursectomy, AT debridement, calcaneal resection, rasp, AT insertion and repair, 5.5 mm suture anchors	PWB in a brace at initial 4w, then WB in a brace, then ROM and brace removal, then heel raise exercise	47 ± 7	92 ± 4	VAS: Pre‐OP: 9 ± 0.9	‐	‐	3 cases of wound infection
											Post‐OP: 2 ± 0.59			
Allam et al. [4]	O	21	‐	Prone	‐	Midline incision/AT split	AT debridement, bursectomy, calcaneal resection, AT augmentation/osteotome, suture anchors, fluoroscopy	Discharge at 48 h, then PWB at 4w, then FWB at 8w, and immobilization for 6w if greater than 50% of the tendon insertion	56	89.5	12 excellent, 8 good, 1 fair	‐	‐	2 cases of surgical wound infection
Mir et al. [40]	O	29	X‐ray	‐	Regional	Lateral	Bursectomy, posterosuperior calcaneal resection, AT debridement, rasp/rongeur	NWB in short‐leg cast for 4w, then PWB as tolerated, then patients can walk without aids at 6–8w, and gastroc soleus strengthening exercises were arranged during rehabilitation	54 (39–79)	86 (60–97)	F‐P angle	‐	‐	5 cases of superficial infection
														1 case of local betadine allergy
Hardy et al. [19]	O	46	MRI	Prone	‐	Posteromedial, midline incision	AT detachment, posterosuperior calcaneal resection, bursectomy, AT debridement	1. Only debridement group: PWB in a walking boot for 6w	62.2 ± 11.7	93.7 ± 7.3	VISA‐A: Post‐OP: 92 ± 5.6	‐	‐	1 case of superficial phlebitis
								2. AT detached and reattached group: NWB for 6w, then gradually walking in a walking boot and regaining ROM			ATRS: Post‐OP: 89.3 ± 4.7			1 case of cyst at middle of the tendon
Barsan et al. [6]	E	27	X‐ray	Prone	Regional, general	Two‐portal	Bursectomy, calcaneoplasty/endoscopic shaver	‐	67	95	‐	‐	‐	2 superficial surgical site infections
Xia et al. [66]	O	22	X‐ray	Prone	‐	Midline incision/AT split	AT detachment, bursectomy, posterosuperior calcaneal osteotomy/oscillating saw and rogue, 2 suture anchors	NBW in cast for 2w, then PWB in a walking boot for the next 4w, then shoes were allowed and rehabilitation	39.3 ± 19.5	83.0 ± 30.7	VAS: Pre‐OP: 7.8 ± 2	‐	‐	2 cases of delayed wound healing
			Ultrasound								Post‐OP: 1.8 ± 2.7			1 case of sensation loss over the heel
											SF‐36 (PCS): Pre‐OP: 36.1 ± 11.9			
											Post‐OP: 44.0 ± 11.3			
											SF‐36 (MCS): Pre‐OP: 48.6 ± 12.9			
											Post‐OP: 52.1 ± 10.8			
Mansour et al. [39]	E	17	X‐ray	Prone	Spinal	Two‐portal/three‐portal	retrocalcaneal space dissection, 4 mm, 30° endoscope, Bursectomy, calcaneoplasty, fluoroscopic guidance, 4‐mm shaver	Cast removed after 1w, then FWB as tolerated	63.4	88.5	‐	8w	12w	3 cases of paraesthesia
														3 cases of scar tenderness
	O	17				Lateral	Calcaneal resection, 0.5 in. curved osteotome, smoothing with a rongeur and curette	Elevation of the foot for the first 1w, PWB for the first 2w, then cast is removed at 2w and try ROM, then FWB at 3w	61.1	80.6	‐	‐	‐	1 case of superficial wound infection
Ewais et al. [13]	E	26	X‐ray	Prone	General, spinal	Two‐portal	Calcaneoplasty, bursectomy/4 mm arthroscope, 4 mm shaver, fluoroscopic guidance	Elevation of the foot and ROM training at the first week, then PWB at 2w, then FWB at 3w	63.3 ± 11.9	86.8 ± 10.1	17 excellent	‐	12w	One case of Achilles tendon rupture 3w after surgery, and transfer to open procedure
			MRI								7 good			
											2 poor (from the same person)			
Aldahshan et al. [1]	E	50	X‐ray	Prone	General, spinal	Two‐portal	Bursectomy, calcaneoplasty/4.5 mm 30° arthroscope, 5‐mm full endoscopic shaver	Gradually increase WB with crutches at first 2w and ROM training	56.8 (43–70)	88.1 (80–95)	VAS: Pre‐OP: 7.9 (6–9)	‐	‐	1 case of superficial infection
											Post‐OP: 2.1 (1–4)			
											23 excellent			
											21 good			
											6 fair			
Jiang et al. [23]	O(single‐row)	16	X‐ray	Prone	‐	Lateral	AT detachment, AT debridement, posterosuperior calcaneal resection, double row AT suture/osteotome	NWB with short leg plaster for 6w, then FWB and ROM training after 6w, then normal daily activities usually at 3 m	56.1 ± 4.1	81.3 ± 6.5	VISA‐A: Pre‐OP: 52.6 ± 5.2	‐	‐	2 cases of recurrence
	O(double‐row)	16	MRI				AT detachment, AT debridement, posterosuperior calcaneal resection, single row AT suture/osteotome		59.2 ± 6.7	91.1 ± 4.2	Post‐OP: 84.1 ± 3.9			5 cases of residual heel pain
											VISA‐A: Pre‐OP: 50.6 ± 3.2	‐	‐	Nil
											Post‐OP: 90.6 ± 3.4			
Hong et al. [28]	O	22	X‐ray	Prone	General	Midline incision/AT split	Central splitting, AT detachment and debridement, Calcaneoplasty, bursectomy, single 4.5‐mm (5.5‐mm) suture anchor.	Wearing anterior plaster slab for the first 2w, then FWB with a short walking boot, then transition to conventional shoes at 6w	66.82 ± 15.15	86.77 ± 11.73	VAS: Pre‐OP: 7.23 ± 2.07	‐	‐	1 case of superficial infection
											Post‐OP: 2.59 ± 2.44			1 case of hypertrophic scar
											SF‐36 score			1 case of partial‐thickness AT tear
Jerosch et al. [22]	E	164	X‐ray, MRI	Supine	Epidural, general, local	Two‐portal	Bursectomy, calcaneal resection/4 mm arthroscope, endoscopic shaver	Elevation of the foot at 5–7 d, PWB for the first 2w, then gradually FWB as tolerated, then normal shoes were not allowed in 6w and no athletic activities for at least 12w	‐	‐	Ogilvie–Harris	‐	12w	1 case of superficial infection
											84 excellent			
											71 good			
											5 fair			
											4 poor			
Natarajan et al. [46]	O	46	X‐ray	‐	‐	Lateral	Calcaneal osteotomy, 1/2 curved osteotome, smoothing edges with rasp	‐	58	86 (60–97)	‐	‐	‐	3 cases of superficial wound infection
														4 cases of delayed recovery
														4 cases of recurrence
Ettinger et al. [12]	O	40	X‐ray, MRI	Prone	General	Midline incision/AT split	AT central detachment, AT debridement, AT reinsertion by one single‐anchor, 2‐suture anchor or double‐row anchor	PWB in a walking boot for 6w, then no sport for at least 4–6 m, and thrombosis prophylaxis with heparin before FWB	59.4 ± 18.4	86.5 ± 12.7	FAOS	14.5 ± 17.6w (2–82)	22.7 ± 13.4w (7–58)	1 case of haematoma, who need secondary surgery
											SF‐36			2 cases of DVT
											Pain (NRS)			3 cases of superficial wound infection
											27 good			2 cases of painful scar
Lin et al. [32]	O	44	‐	‐	‐	Lateral	4 cm incision, Bursectomy, AT detachment, AT debridement, Calcaneoplasty	Movement in a walking boot for 6w, then rehabilitation with ROM and calf muscle strengthening	43.5	86.5	VAS: Pre‐OP: 7.2	‐	‐	3 cases of delayed wound healing
											Post‐OP: 1.7			
											SF‐36			
							(45° posteroinferiorly), two 5.0‐mm suture anchors							
Greenhagen et al. [15]	O	30	X‐ray	Prone	‐	Midline incision/AT split	AT detachment, AT debridement, Calcaneoplasty, AT reattachment	NWB with compressive dressing for 1w, then NWB with CAM boot, then BW as tolerated at 3w, then transition to normal shoes and physical therapy as needed	56.57 ± 14.02	91.67 ± 10.35	19 excellent	‐	‐	Nil
											9 good			
											1 poor			
Kaynak et al. [24]	E	30	X‐ray, MRI	Prone, supine	General	Two‐portal	Bursectomy, calcaneal resection, shavers and burrs, fluoroscopic guidance	ROM training was allowed at first day, then PWB as tolerated at 3 d with crutches, then FWB at 2w	52.6 (24–75)	98.6 (90–100)	‐	6w	12w	Nil
Wu et al. [65]	E	25	X‐ray, MRI	Prone	Spinal	Three‐portal	4‐mm 30° endoscope, Bursectomy, Calcaneoplasty, AT debridement, burr and shaver	Elevation of the foot with ROM training for the first week, then PWB at 2w, then FWB at 3w	63.3 ± 11.9	86.8 ± 10.1	Ogilvie–Harris score:	‐	‐	Nil
											15 excellent			
											7 good			
											1 fair			
											2 poor			
Kondreddi et al. [26]	E	25	X‐ray, Ultrasound	Prone, semipro‐ne	Spinal	Two‐spinal	30° arthroscope, Bursectomy, Calcaneoplasty, AT debridement, arthroscopic 4 mm shaver and burr	NWB for first 2w, then walking with modified footwear for 2 m, then transition to normal footwear	57.9 ± 6.2	89.1 ± 5.3	Maryland scores Post‐OP: 90.28 ± 5.77	‐	‐	1 case of superficial wound infection
											Excellent: 16 Good: 6			2 cases of sural neuropathy
											Fair: 3			1 case of DVT
Maffulli et al. [35]	O	30	X‐ray	Prone	General	Cincinnati incision.	Bursectomy, Calcaneal resection, AT debridement	Discharge 8 h after surgery, then PWB as tolerated with crutches, then cast removed at 2w, then gradually increase weight till FWB, then start rehabilitation	‐	‐	VISA‐A: Pre‐OP: 62 ± 2.2 (48–74)	‐	‐	2 cases of superficial infection
											Post‐OP: 88 ± 2.2 (77–95)			
Anderson et al. [5]	O (lateral)	35	X‐ray	Prone	‐	Lateral	Bursectomy, calcaneal osteotomy/0.5 in. curved osteotome, rongeur, rasp, suture anchors, AT insertion, repair	Wearing cast for 4w, then followed by a CAM boot for 4–6w, and BW as tolerate at 6–8w with rehabilitation twice a week for 4w	54 (10–72)	86 (10– 100)	VISA‐A (both group) Pre‐OP: 48.8 (10–72)	6.4 m (4–20)	6.5 m (4–27)	2 cases of superficial wound infection
	O (midline)	31				Midline	AT split, bursectomy, calcaneal osteotomy/0.5 in. curved osteotome, rongeur, rasp, suture anchors, AT insertion, repair		43 (10–67)	81 (10– 100)	Post‐OP: 83.7 (10–100)	4.1 m (3‐13)	5.4 m (4–21)	2 cases of superficial wound infection
											Physical SF‐36: Midline 52 (3–13) Lateral 49 (34–63)			1 case hypertrophic scar
											Mental SF‐36: Midline: 54 (22–61) Lateral 53 (20–59)			1 case of AT rupture
Ortmann et al. [51]	E	30	X‐ray, MRI	Supine	General, regional	Two‐portal	4 or 2.7 mm arthroscopy, calcaneal resection, Bursectomy, 4 mm burr and shaver, 18‐gauge needle insertion, fluoroscopic guidance	NWB in the splint for 10–14 d, then transition to a walking boot for 2–3w, then normal walking with normal shoes at 4w, then athletic activities at 6–12w	62 ± 12.7	97 ± 6.1	Excellent: 26	8w	12w	1 case of AT rupture
									(36–77)	(78–100)	Good: 3 Moderate: 1			1 case of residual pain and swelling
Scholten et al. [57]	E	39	X‐ray	Prone	General, regional	Two‐portal	4.5 mm 30° or 70° arthroscopy, 5.0‐mm full radius resector, calcaneal resection, Bursectomy, shaver and burr/acromionizer	BW as tolerated after surgery and ROM training	‐	‐	Ogilvie–Harris	5w (10d to 6m)	11w	1 case of delayed recovery
											Excellent: 24		(6w to 6m)	
											Good: 6			
											Fair: 4			
											Poor: 2			
Brunner et al. [8]	O	39	X‐ray	Supine	‐	Medial/lateral	Bursectomy, Posterosuperior calcaneal resection, AT debridement, bone anchors, Dacron suture, fluoroscopy	NWB for 4w in cast, then gradually increasing weight in the following 2w, then exchange cast to walking boot at 6w for 2w, then physical therapy	54	86 (55– 100)	SF‐36 (Physical): 46 (36–59)	‐	‐	2 cases of superficial wound infection
											SF‐36 (Mental): 47 (24–61)			1 case of delayed recovery
											30 good			
											3 fair			
											6 poor			6 cases of persistent pain
Maffulli et al. [38]	O	21	X‐ray	Prone	General	Medial	Bursectomy, Calcaneal resection, AT debridement	Discharge 8 h after surgery, then PWB as tolerated with crutch, then cast removed at 2w, then gradually increasing weight til FWB, then started athletic activities at 20–24w	‐	‐	VISA‐A: Pre‐OP: 63.8 (51–78)	‐	‐	2 cases of superficial infection
											Post‐OP: 86.4 (78–94)			
											Excellent: 11			
											Good: 5			3 cases of hypersensitive wound
											Moderate: 5			1 case of hypertrophic scar
Leitze et al. [31]	E	33	X‐ray	Supine	General	Two‐portal, Single‐portal with fluoroscopic	4 mm 30° arthroscopy, Bursectomy, Calcaneal osteotomy, 4‐mm hooded burr and shaver, AT debridement, suture anchors	NWB for 2w, then walking with modified footwear for the following 3 m, then transition to normal shoes	61.8 ± 12.9	87.5 ± 15.0	Scope: Maryland	‐	‐	1 case of wound infection
											86 ± 17			
											19 excellent			
											5 good			
											3 fair			3 cases of paraesthesia
											3 poor			2 cases of scar tenderness
	O	17				Medial, lateral	Calcaneal osteotomy, AT debridement, Retrocalcaneal decompression		58.1 ± 17.6	79.3 ± 19.0	3 poor	‐	‐	3 cases of symptoms recurrence
														2 cases of superficial wound infection
														3 cases of paraesthesia
														3 cases of scar tenderness
Calder et al. [9]	O	52	‐	‐	‐	Midline incision/AT split	Bursectomy, AT debridement, calcaneal osteotomy	PWB for 10–14 d with crutch, then active ROM training and gradually increased WB till FWB	‐	‐	‐	‐	‐	3 cases of superficial wound infection
														2 cases of AT avulsion

Abbreviations: AAS, ankle activity score; ATRS, Achilles tendon total rupture score; C‐L angle, Chauveaux–Liet angles; d, day(s); E, endoscopic; EQ‐5D, EuroQol‐5 Dimension; FAOS, Foot and Ankle Outcome Score; FFI, Foot Function Index; F‐P angle, Fowler & Philip angle; FWB, full‐weight‐bearing; JSSF, Japanese Society for Surgery of the Foot; M, month(s); MCS, mental component scores; NRS, Numerical Rating Scale; NWB, no‐weight‐bearing; O, open; PCS, physical component scores; PWB, partial weight‐bearing; partial‐weight‐bearing; ROM, range of motion; RTC, time (in months) to return to at least 1 min of official match play post‐operatively; RTP, rate of athlete participation in at least two full seasons post‐operatively; SF‐12 MCS, 12‐Item Short Form Survey (Mental Component Summary); SF‐12 PCS, 12‐Item Short Form Survey (Physical Component Summary); SF‐36, short form‐36; VAS, visual analogue scale; VISA‐A, The Victorian Institute of Sport Assessment‐Achilles; W, week(s); WB, weight‐bearing; yr, year(s)

### Surgical techniques

All included studies required patients to have undergone at least 6 months of unsuccessful conservative management prior to surgical intervention. Although various surgical techniques for IAT have been described, the present study specifically compared the open and endoscopic techniques.

Regarding open surgery, four approaches were reported: midline with AT split [4, 5, 7, 9, 12, 13, 16, 17, 18, 20, 29, 41, 59, 67], which was the most commonly reported method; the medial approach [8, 32, 35], the lateral approach [5, 8, 15, 24, 31, 32, 33, 35, 40, 41, 47, 55]; and Cincinnati incision, which was only mentioned by Maffulli et al. [36, 37, 39]. The most commonly performed procedures in the open surgical group included bursectomy, calcaneoplasty, AT reattachment and AT debridement.

Regarding endoscopic surgery, the two‐portal approach was the most common, used in 13 studies [1, 6, 12, 14, 23, 25, 27, 32, 40, 42, 52, 55, 58]. Wu et al. [66] employed a three‐portal method, incorporating proximal posterolateral, distal posterolateral and distal posteromedial portals. Mansour [40] used both two‐ and three‐portal approaches, depending on the specific case. Leitze et al. [32] employed both two‐ and single‐portal techniques with fluoroscopic guidance.

The prone position was the most commonly used patient positioning method, which was reported in 27 studies [1, 4, 5, 6, 7, 12, 13, 14, 16, 17, 18, 20, 24, 25, 27, 29, 35, 36, 37, 39, 40, 42, 46, 55, 58, 59, 66, 67]. However, only four studies [8, 25, 32, 52] employed the supine position. Kaynak [25] employed both supine and prone positions, depending on the specific surgical case, whereas Kondreddi et al. [27] used a combination of prone and semiprone positions, also depending on the specific case.

### Quality assessment and risk of bias

The evidence provided by the studies, as determined using the Oxford Centre for Evidence‐Based Medicine (2011) criteria, was predominantly classified as Level III [5, 6, 7, 24, 27] or Level IV [1, 4, 5, 6, 7, 9, 12, 13, 14, 15, 16, 17, 18, 20, 23, 24, 25, 27, 29, 31, 33, 36, 39, 41, 42, 47, 52, 55, 58, 66, 67]. Only three studies—Thiounn et al. [64], Mansour [40], and Leitze et al. [32]—provided Level II evidence. Regarding study quality, the majority of the studies were rated as low or very low, with only Thiounn et al. [64] and Leitze et al. [32] receiving a moderate quality rating. Both the open and endoscopic surgical groups exhibited substantial heterogeneity (*I* ^2^ = 92% and 87%, respectively). However, heterogeneity between the two groups was low (*I* ^2^ = 0%).

Regarding the risk of bias, 26 studies were rated as having a high risk of bias based on the MINORS [1, 4, 6, 9, 13, 14, 15, 16, 17, 18, 20, 23, 24, 25, 29, 33, 35, 36, 41, 42, 46, 47, 58, 59, 66, 67]. The average MINORS score was 18.6 for comparative studies and 10.8 for single‐group studies. Detailed scoring information is provided in Table 3.

**Table 3:** Methodological Index for Non‐Randomized Studies for risk of bias.

Source	Clear stated aim	Consecutive patients	Prospective collection of data	End point appropriate to the study aim	Unbiased evaluation of end point	Follow‐up period appropriate to the aim of the study	Loss to follow‐up not exceeding 5%	Prospective calculation of the study size	Adequate control group	Contemporary groups	Baseline equivalence	Adequate statistical analyses	Total score
Thiounn et al. [63]	2	2	2	2	2	1	0	0	2	2	2	2	19/24
Lugani et al. [34]	2	2	0	2	1	2	2	0	‐	‐	‐	‐	11/16
Nakajima et al. [45]	2	2	0	2	1	2	2	0	‐	‐	‐	‐	11/16
Scoott et al. [58]	2	1	0	2	1	2	2	0	‐	‐	‐	‐	10/16
Maffulli et al. [36]	2	2	2	2	1	2	2	0	‐	‐	‐	‐	13/16
Baumbach et al. [7]	2	2	0	2	1	2	2	0	‐	‐	‐	‐	12/16
Lee et al. [30]	2	2	0	2	1	2	2	2	‐	‐	‐	‐	13/16
Greiner et al. [16]	2	1	0	2	1	2	2	0	‐	‐	‐	‐	10/16
Cusumano et al. [11]	2	1	0	2	1	2	2	0	2	2	2	1	17/24
Pi et al. [54]	2	1	0	2	1	2	1	0	2	2	2	1	16/24
Mishra et al. [41]	2	1	2	2	1	2	1	0	‐	‐	‐	‐	11/16
Ge et al. [14]	2	1	0	2	1	2	2	0	‐	‐	‐	‐	10/16
Yasin et al. [17]	2	1	0	2	1	2	2	0	‐	‐	‐	‐	10/16
Allam et al. [4]	2	2	0	2	1	2	2	0	‐	‐	‐	‐	11/16
Mir et al. [40]	2	2	0	2	1	2	2	0	‐	‐	‐	‐	11/16
Hardy et al. [19]	2	1	1	2	1	2	2	0	‐	‐	‐	‐	11/16
Basran et al. [6]	2	1	2	2	2	1	1	0	‐	‐	‐	‐	11/16
Xia et al. [66]	2	2	0	2	2	2	0	0	–	‐	‐	‐	10/16
Mansour et al. [39]	2	1	0	2	1	2	2	0	2	2	2	1	17/24
Ewais et al. [13]	2	1	0	2	1	2	2	0	‐	‐	‐	‐	10/16
Aldahshan et al. [1]	2	1	2	2	1	2	1	0	‐	‐	‐	‐	11/16
Jiang et al [23]	2	2	0	2	1	2	2	0	‐	‐	‐	‐	11/16
Hong et al. [28]	2	1	2	2	1	2	1	0	‐	‐	‐	‐	11/16
Jerosch et al. [22]	2	1	0	2	1	2	2	0	‐	‐	‐	‐	10/16
Natarajan et al. [46]	2	1	0	2	1	2	1	0	‐	‐	‐	‐	9/16
Ettinger et al [12]	2	1	0	2	1	1	1	0	‐	‐	‐	‐	8/16
Lin et al. [32]	2	1	0	2	1	1	1	0	‐	‐	‐	‐	8/16
Greenhagen et al. [15]	2	2	0	2	1	2	2	0	‐	‐	‐	‐	10/16
Kaynak et al. [24]	2	2	0	2	1	2	2	0	‐	‐	‐	‐	11/16
Wu et al [65]	2	2	0	2	1	2	2	0	‐	‐	‐	‐	11/16
Kondreddi et al. [26]	2	2	2	2	1	1	2	0	‐	‐	‐	‐	12/16
Maffulli et al [35]	2	2	0	2	1	2	0	0	‐	‐	‐	‐	9/16
Anderson et al. [5]	2	2	0	2	2	2	2	0	‐	‐	‐	‐	12/16
Ortmann et al. [51]	2	2	1	2	2	2	1	0	‐	‐	‐	‐	12/16
Scholten et al. [57]	2	2	0	2	1	2	2	0	‐	‐	‐	‐	11/16
Brunner et al. [8]	2	2	0	2	2	2	2	0	‐	‐	‐	‐	12/16
Maffulli et al. [38]	2	2	2	2	2	2	2	0	‐	‐	‐	‐	14/16
Leitze et al. [31]	2	2	2	2	2	2	2	2	2	2	2	2	24/24
Calder et al. [9]	2	2	0	2	0	2	2	0	‐	‐	–	‐	10/16

To evaluate potential publication bias, funnel plot analysis was conducted for the primary outcome. The funnel plot showed approximate symmetry (Supplementary data Figure S1).

### Outcome measures

Several outcome measures were employed in this study to evaluate surgical results. Among them, the AOFAS score was the most commonly reported, appearing in 29 studies [1, 4, 5, 6, 8, 12, 13, 14, 15, 16, 17, 18, 20, 24, 25, 27, 29, 31, 32, 33, 35, 40, 41, 42, 47, 52, 55, 66, 67]. Pi et al. [55] reported only post‐OP outcomes, whereas the remaining 28 studies provided both pre‐OP and post‐OP scores. In these 28 studies, the mean pre‐OP score was 56.07, and the mean post‐OP score was 89.17.

A comparison by surgical approach revealed that the open surgical group had a mean pre‐OP score of 54.15 and a mean post‐OP score of 88.35, whereas the endoscopic surgical group had a mean pre‐OP score of 61.29 and a mean post‐OP score of 91.41. However, four studies [4, 6, 33, 40] reported only mean scores without SD or range data, and two studies [8, 47] lacked pre‐OP SD or range data, further limiting our comparative analysis.

Additionally, five studies [1, 5, 25, 35, 41] provided ranges only for pre‐ and post‐OP scores. To enable inclusion in the analysis, we converted the reported ranges into SDs by using the method described by Hozo et al. [21]. Thus, 22 studies [1, 5, 12, 13, 14, 15, 16, 17, 18, 20, 24, 25, 27, 29, 31, 32, 35, 41, 42, 52, 66, 67] were included in the final analysis.

**Figure 2:**
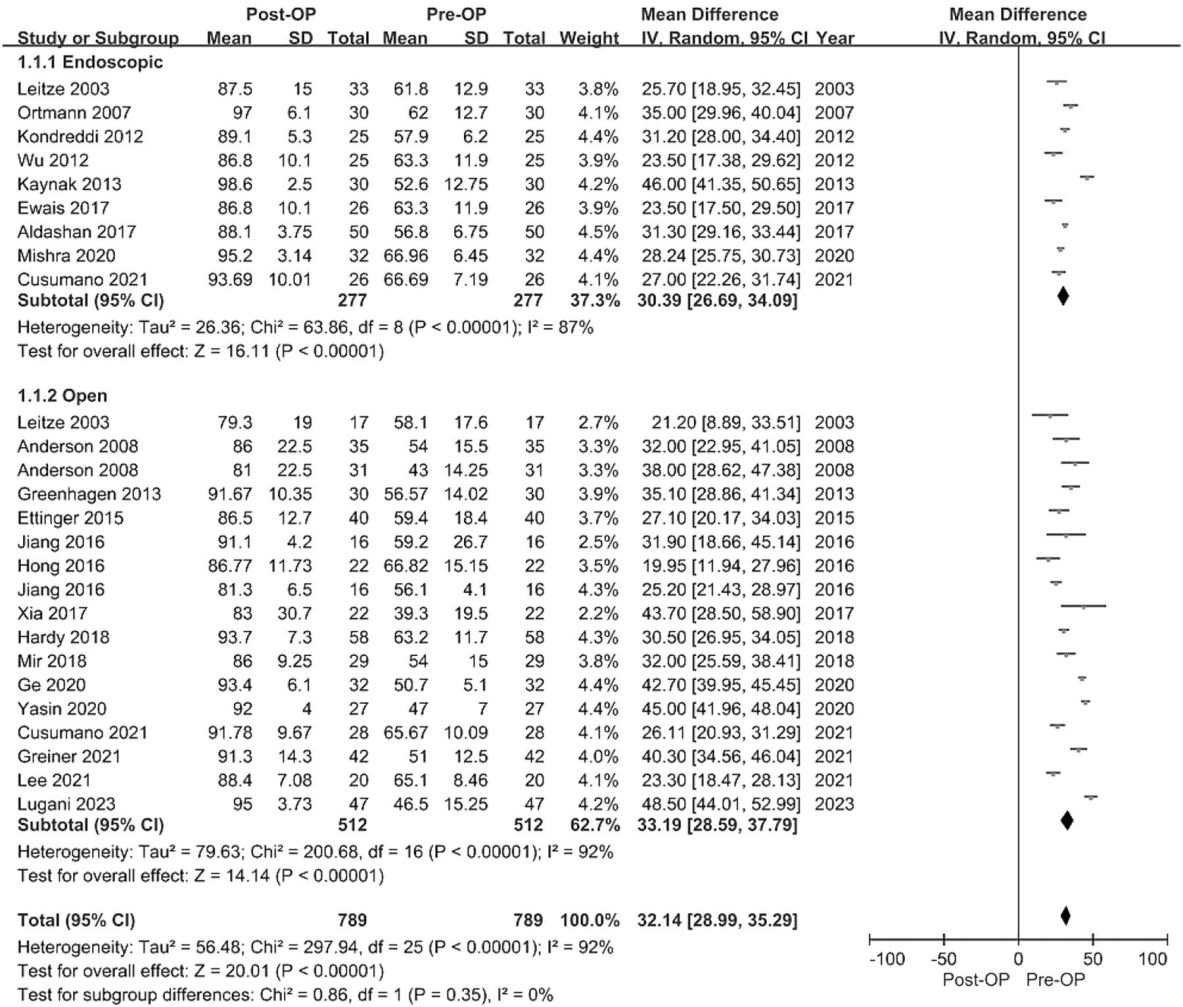
Forest plot of change between pre‐OP and post‐OP in open and endoscopic surgical intervention. CI, confidence interval; IV, intravenous; post‐OP, post‐operative; pre‐OP, preoperative; SD, standard deviation.

The meta‐analysis results revealed significant improvements in AOFAS scores for both groups, as illustrated in Figure 2. Specifically, the score for open surgery was 33.19 (95% CI: 28.59–37.79, *p* < 0.001, *I* ^2^: 92%), and that for endoscopic surgery was 30.39 (95% CI: 26.69–34.09, *p* < 0.001, *I* ^2^: 87%).

Despite the observed improvements in functional scores, no significant difference was observed between the open and endoscopic surgical groups (*p* = 0.35), with minimal heterogeneity (*I* ^2^ = 0%).

The VISA‐A score [22] was reported in 11 studies [5, 7, 15, 20, 24, 35, 36, 37, 39, 46, 64], which provided a mean pre‐OP score of 44.14 ± 14.44 and a mean post‐OP score of 84.98 ± 15.38. The VAS [30] was used in 10 studies [1, 12, 17, 18, 29, 33, 46, 55, 59, 67], which reported a mean pre‐OP score of 7.35 ± 1.30 and a mean post‐OP score of 1.53 ± 1.49. Less frequently reported outcome measures included the 12‐item short‐form survey score [63], Fowler–Philip angle [49], AT rupture score [48], University of Maryland foot score [57] and Ogilvie–Harris score [51]. Some studies also reported patient satisfaction rates, which were divided into four categories, namely excellent, good, fair and poor; the majority of the patients reported excellent satisfaction (60.15%), followed by those reporting good (28.78%), fair (6.46%) and poor (4.06%) satisfaction [1, 4, 8, 14, 16, 23, 27, 31, 32, 39, 42, 52, 58, 66]. A detailed summary of individual study findings is presented in Table 2.

### Sensitivity analysis

**Figure 3:**
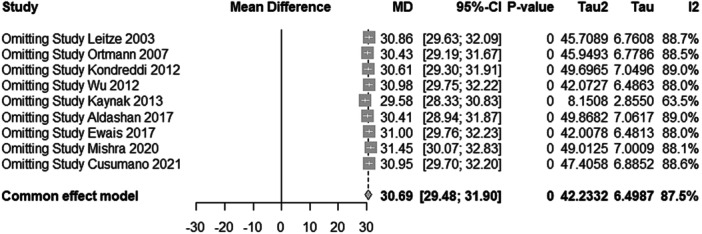
One‐study‐removed sensitivity analysis for functional improvement in the endoscopic surgery group. CI, confidence interval.

**Figure 4:**
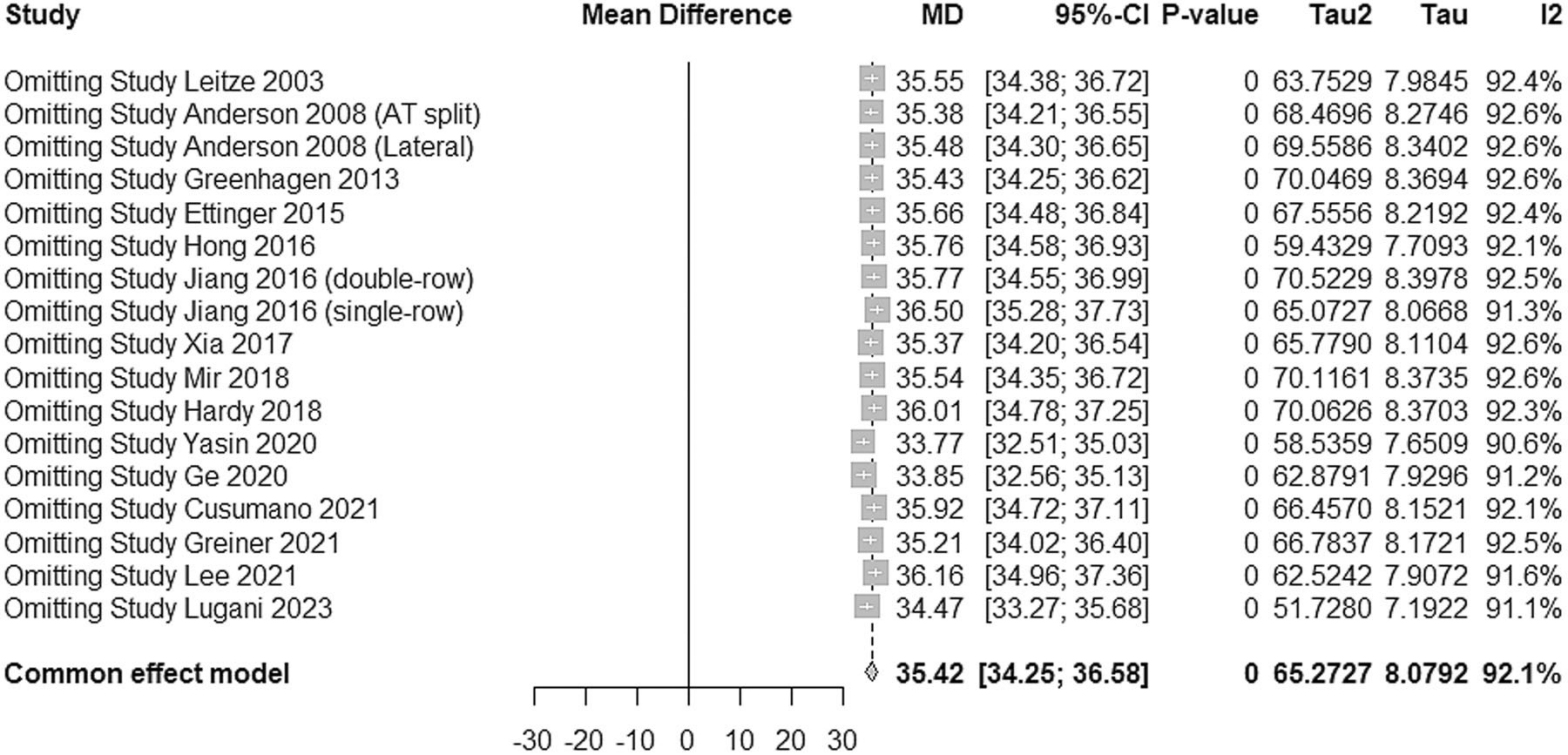
One‐study removed the sensitivity analysis for functional improvement in the open surgery group. CI, confidence interval.

A one‐study removed sensitivity analysis was performed for both surgical groups to assess the robustness of pooled effect estimates and the impact of individual studies on heterogeneity. In the endoscopic group, the pooled mean difference for functional improvement was 30.69 (95% CI: 29.48–31.90, *p* < 0.001), with substantial heterogeneity (*τ* ^2^ = 42.23, *I* ^2^ = 87.5%). Notably, exclusion of the study by Kaynak et al. reduced the heterogeneity to a moderate level (MD = 29.58, 95% CI: 28.33–30.83, *τ* ^2^ = 8.15, *I* ^2^ = 63.5%), as illustrated in Figure 3 [25]. In contrast, the open surgery group exhibited consistently high heterogeneity across all iterations, with a pooled mean difference of 35.42 (95% CI: 34.25–36.58, *p* < 0.001, *τ* ^2^ = 65.27, *I* ^2^ = 92.1%), and no single study removal markedly altered the heterogeneity, as shown in Figure 4.

### Complications, surgical failure and return to activity

Surgical failure was defined as symptom recurrence, AT rupture, or the need for secondary surgery. In the open surgical group, 13 out of 988 ankles (1.3%) experienced surgical failure, compared with only 2 out of 637 ankles (0.3%) in the endoscopic surgical group. This difference was statistically significant (*p* < 0.001).

Complications, including infection, paraesthesia, scar tenderness and delayed recovery, are summarized in Table 2. A total of 122 complications were reported in 988 ankles in the open surgical group (12.4%), whereas 34 complications were reported in 637 ankles in the endoscopic surgical group (5.3%); this difference was statistically significant (*p* < 0.001).

**Figure 5:**
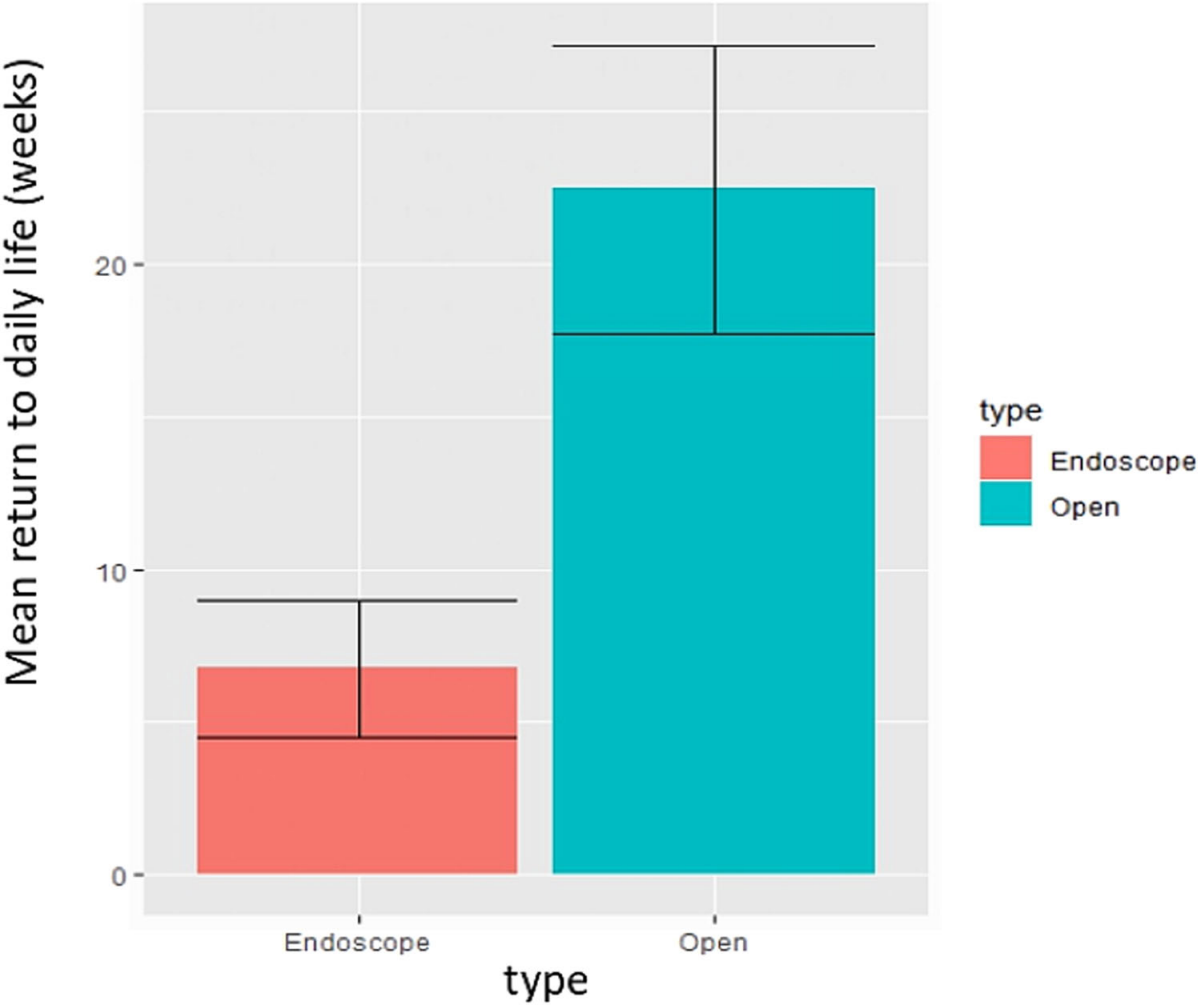
Mean time (weeks) return to daily life post‐operatively after open and endoscopic surgery.

**Figure 6:**
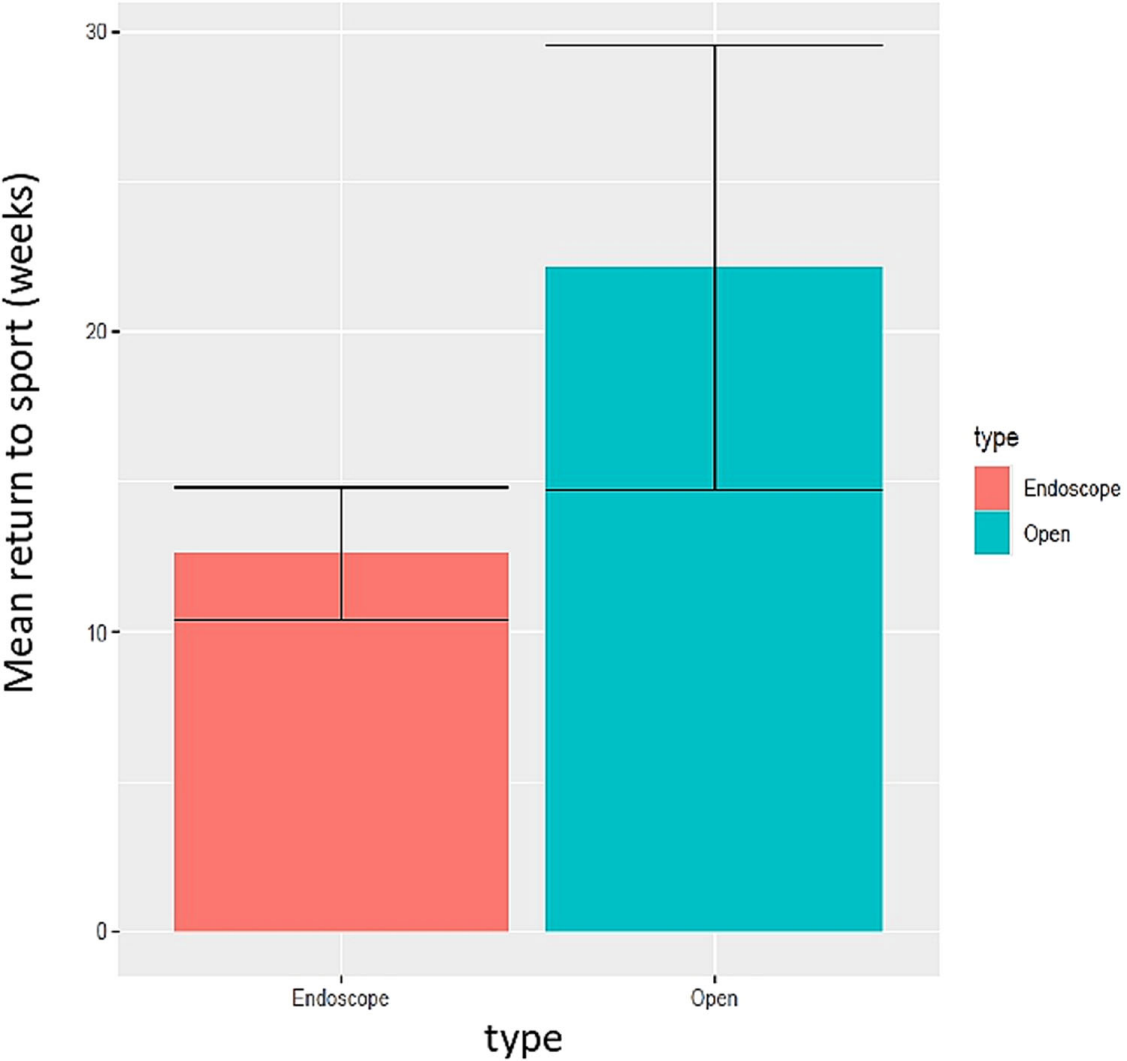
Mean time (weeks) return to sport post‐operatively after open and endoscopic surgery.

The mean time (±SD) to return to daily activities and mean time (±SD) to return to sports post‐operatively were reported in 6 studies [5, 13, 25, 40, 52, 58] and 10 studies [5, 13, 14, 23, 25, 31, 40, 46, 52, 58], respectively (Figures 5 and 6). The mean time to return to daily activities was 22.45 ± 4.74 weeks in the open surgical group and 6.75 ± 2.25 weeks in the endoscopic surgical group, and the mean time to return to sports was 22.13 ± 7.42 weeks in the open surgical group and 12.63 ± 2.2 weeks in the endoscopic surgical group; the differences were also statistically significant (*p* < 0.001).

Patients undergoing endoscopic surgery returned to daily activities within 6.75 weeks (~1.5 months). However, the time to return to sports was 12.63 weeks (~3 months), significantly longer than the time to return to daily activities.

### Subgroup analysis: Highly active populations

Table 4 presents data from six studies [20, 25, 29, 31, 46, 66] that specifically investigated athletes or individuals engaged in high‐intensity physical activities. Among these studies, the proportion of highly active patients undergoing surgical treatment for IAT ranged from 17.9% to 100% of the total patient population. The participants comprised professional, semiprofessional and recreational athletes across various sports, including soccer, running and basketball. Endoscopic surgery [25, 46, 66] had a shorter time to return to sports (3.5–4.5 months), with some athletes returning to team training by 6 weeks and competition by 3 months. By contrast, open surgery [20, 29, 31] had a longer time to return to sports (5–7.5 months), with Lee et al. [31] reporting an average time of 7.45 months (range, 4–18 months).

**Table 4:** Summary of studies reporting outcomes of physically active population.

	Patients proportion	Surgical approach	Mean RTS (weeks)	Outcome measure	Key findings
Nakajima [45]	22 athletes (50%)	E	18	‐	Reported RTS but lacked subgroup analysis for athletes
Lee et al. [30]	20 professional athletes with 13 soccer players (100%)	O	30 (16–72)	AOFAS	Higher BMI delayed RTS (*p* = 0.005); 60% sustained competitive play for ≥2 seasons
				Pre‐OP: 65.1 ± 8.4	
				Post‐OP: 88.4 ± 7.08	
Hardy et al. [19]	26 in high‐intensity sports (47.8%)	O	18	71.7% of patients returned to their preoperative or higher activity level.	Early weight‐bearing promoted faster functional recovery
				82.6% of patients had no limitations during physical activity.	
Lai et al. [28]	10 recreational athletes (45.5%)	O	21 (8–48)	VAS: Pre‐OP: 7.23	70% returned to pre‐injury sports level; longer RTS than endoscopic surgery
				Post‐OP: 2.59	
Kaynak et al. [24]	5 professional athletes (17.9%)	E	12	‐	RTS faster than open surgery; team training resumed at 6 weeks
Wu et al. [65]	Younger, active patients (mean age: 27.7)	E	12–24		Faster pain relief and function recovery than open surgery

Abbreviations: AOFAS, American Orthopaedic Foot and Ankle Society; BMI, body mass index; E, endoscopic; O, open; Post‐OP, post‐operative; Pre‐OP, preoperative; RTS, return to sport; VAS, visual analogue scale.

Both surgical techniques had significant improvements. Endoscopic surgery has faster pain relief (AOFAS score increased from 62–65 to 90–98) and higher satisfaction rates (87%–97%), as presented in Table 2.

### Rehabilitation protocol

Among the 39 studies reviewed, 36 documented rehabilitation programmes [1, 4, 5, 7, 8, 9, 12, 13, 14, 15, 16, 17, 18, 20, 23, 24, 25, 27, 29, 31, 32, 33, 35, 36, 37, 39, 40, 41, 42, 46, 52, 55, 58, 59, 66, 67]. In the open surgical group, patients were typically restricted from weight‐bearing for 2–6 weeks by using a cast or splint. By contrast, endoscopic surgery allowed for a significantly shorter recovery period, with earlier weight‐bearing (initiated by Weeks 2–3); rehabilitation programmes emphasized early ROM exercises, proprioception training, and gastrocnemius complex strengthening. Following endoscopic procedures, athletes could commence light jogging by Weeks 10–12, and full return to sports was achieved within 12–18 weeks. In open surgery cases, rehabilitation was prolonged, with full return to sports requiring 20–30 weeks.

Early progressive loading within the first 6 weeks was associated with improved rates of return to sports, particularly in athletes undergoing endoscopic surgery. A higher body mass index (BMI) was associated with delayed recovery, emphasizing the importance of preoperative conditioning [31]. Athletes were generally cleared for full competition once they achieved 90% strength symmetry and pain‐free plyometric performance. These findings underscore the importance of early rehabilitation protocols and individualized recovery strategies in optimizing functional outcomes and return‐to‐sports timelines, particularly for high‐performance athletes.

## DISCUSSION

IAT is a degenerative condition that affects the AT insertion; it often coexists with Haglund's deformity. Although conservative treatment remains the primary management approach, surgical intervention—either open or endoscopic—is considered when nonoperative measures fail. This systematic review and meta‐analysis compared the long‐term outcomes, complication rates, and recovery times of open versus endoscopic surgery for IAT. A total of 39 observational studies with 1,559 patients and 1,625 procedures were analyzed to provide findings that can guide surgical decision‐making.

The primary finding of this systematic review and meta‐analysis is that long‐term outcomes based on AOFAS scores did not differ significantly between the endoscopic and open surgical groups. However, the endoscopic surgical group exhibited a lower complication rate and a faster return to activities compared with the open surgical group.

Alessio‐Mazzola et al. [2] conducted a systematic review of 35 studies published between 1995 and 2020 and revealed that endoscopic surgery was associated with greater postoperative improvement in AOFAS scores, a lower complication rate, a reduced failure rate, and a shorter mean time to return to daily activities or sports compared with open surgery. However, in the present study, the endoscopic surgical group demonstrated favourable trends in complication rates (5.3% vs. 12.4%), surgical failure (0.3% vs. 1.3%), and recovery time, but the improvement in AOFAS scores did not differ significantly between the open (mean improvement: 33.19) and endoscopic (mean improvement: 30.39) surgical groups (*p* = 0.35). The accelerated recovery in the endoscopic group may be attributed to reduced soft tissue trauma and smaller incisions. This shortened recovery timeline may be particularly beneficial for patients engaged in high‐intensity sports, where adequate tendon healing and timely return to activity are critical to prevent re‐injury [20, 25, 29, 31, 46, 66].

To assess the robustness of our findings, we performed a one‐study‐removed sensitivity analysis for both surgical groups. In the endoscopic group, when Kaynak et al. were excluded, *I* ^2^ decreased markedly (*I* ^2^ from 87.5% to 63.5%), highlighting the impact of study‐level differences on pooled outcomes [25]. By contrast, the open surgery group showed persistently high heterogeneity (*I* ^2^ > 91%) regardless of which study was excluded, likely reflecting procedural variability. These findings highlight the need for more standardized techniques and reporting in future research.

However, the improvement in AOFAS scores did not differ significantly between the open (mean improvement: 33.19) and endoscopic (mean improvement: 30.39) surgical groups (*p* = 0.35).

Comparative studies conducted by Thiounn et al. [64], Cusumano et al. [12], Pi et al. [55], Mansour [40] and Leitze et al. [32] have also reported no significant difference between open and endoscopic surgery regarding outcome measures, including AOFAS and VISA‐A scores. However, the endoscopic approach was associated with a lower complication rate.

In contrast to the systematic review by Alessio‐Mazzola et al. [2], the present study focused specifically on comparing open and endoscopic surgical techniques. To improve consistency and minimize short‐term or underpowered data, we applied relatively stricter inclusion criteria, such as requiring studies to include at least 20 patients and a minimum follow‐up of 6 months. While this approach may have excluded some smaller studies with potentially relevant findings, it was intended to prioritize more stable outcome assessments and reduce the risk of short‐term bias.

Endoscopic surgery represents a minimally invasive alternative to open surgery; it provides enhanced visualization through arthroscopy. However, endoscopic techniques require a steeper learning curve, necessitating greater surgical expertise for optimal outcomes. A key determinant of surgical success is the adequate removal of Haglund's deformity, which requires sufficient exposure of the affected area. In open surgery, achieving this exposure often necessitates a larger incision, which increases the risk of wound complications and soft tissue damage. This difference may explain the lower complication rates and faster return to activities observed in patients undergoing endoscopic procedures. Outcomes following surgical treatment for IAT in high‐intensity populations varied considerably depending on the surgical approach employed and patient characteristics. Endoscopic surgery was consistently associated with a shorter time to return to sports (average: 12–18 weeks), whereas open surgery required a longer recovery period of 20–30 weeks, with some cases extending beyond a year. Studies by Kaynak [25] and Wu et al. [66] have reported that highly active individuals undergoing endoscopic surgery could resume team training within 6 weeks post‐operatively, whereas patients undergoing open surgery required a prolonged rehabilitation period, largely due to delayed wound healing and increased ankle joint stiffness. Additionally, a higher BMI was identified as a significant predictor of delayed return to sports (Lee et al. [31]; *p* = 0.005). Ptak et al. [56] also highlighted the importance of preoperative weight optimization in competitive athletes.

Early rehabilitation and progressive weight‐bearing were strongly associated with improved return‐to‐sports rates. Studies have indicated that athletes who engaged in controlled loading and functional training within the first 6 weeks post‐operatively experienced faster functional recovery and sustained long‐term performance. Moreover, Kvist and Kvist [28] demonstrated that immediate post‐operative mobilization helps prevent adhesion formation, preserves tendon gliding, and promotes circulation, ultimately facilitating faster recovery and reducing complications. This rehabilitation strategy helps prevent soft tissue fibrosis, maintain muscle strength, and enhance tendon elasticity, thereby mitigating joint stiffness, a common consequence of prolonged immobilization. By contrast, delayed rehabilitation was associated with prolonged recovery times (≥6 months) and a lower likelihood of returning to high‐level sports. Considering these findings, endoscopic surgery appears to be the preferred approach for athletes requiring a faster return to sports, whereas open surgery should be reserved for severe cases necessitating extensive debridement. Future research should further investigate the influence of sport‐specific demands and the effectiveness of individualized rehabilitation protocols to optimize surgical decision‐making for high‐performance athletes. This study has several strengths, including a diverse patient population from various countries, such as France [20, 64], Germany [7, 13, 23], Turkey [18, 25], Korea [31], Austria [17], Egypt [1, 4, 14, 40], India [6, 27, 42, 47], Singapore [29, 33, 67], China [15, 24, 55, 66], Italy [12, 35, 36, 37, 39, 41], Japan [46], the United States [5, 8, 16, 32, 52, 59], Australia [9] and the Netherlands [58]. This geographic diversity may have enhanced the external validity of the present findings. Additionally, we analyzed 39 observational studies, including 18 studies [7, 12, 13, 15, 16, 17, 18, 29, 31, 35, 36, 37, 39, 42, 46, 55, 59, 64] published between 2003 and 2024, ensuring that our data are up to date. Moreover, statistical analyses and sensitivity analyses were conducted to compare preoperative and post‐operative AOFAS scores, providing a more objective, data‐driven assessment of surgical outcomes. Notably, our subgroup analysis of highly active individuals [20, 25, 29, 31, 46, 66] provides clinically relevant insights into the optimal surgical approach and rehabilitation strategies for IAT management in high‐performance athletes.

This study also has several limitations that should be acknowledged. High‐quality evidence was lacking because only three studies [32, 40, 64] were prospective cohort studies, whereas the remaining studies included 8 prospective case series [6, 27, 29, 35, 36, 37, 39, 42] and 28 retrospective studies [1, 4, 5, 7, 8, 9, 12, 13, 14, 15, 16, 17, 18, 20, 23, 24, 25, 31, 33, 41, 46, 47, 52, 55, 58, 59, 66, 67]. Because of the predominance of retrospective designs, minor complications may have been underreported, and the recorded time to return to activity may not be accurate, potentially influencing complication rates and recovery time analyses. Furthermore, most of the included studies were classified as having Level III and Level IV evidence, with only three [32, 40, 64] classified as prospective cohorts. The overall study quality was predominantly low to very low, except for studies by Thiounn et al. [64] and Leitze et al. [32], which were rated as moderate. These limitations may have introduced selection and information biases, and the interpretation of pooled results should therefore be made with caution, given the generally low level of evidence. Another limitation is the heterogeneity in surgical techniques. The open surgery group included various approaches—such as midline AT–splitting, lateral, medial and Cincinnati incisions—with differing extents of debridement and reattachment. The endoscopic group also varied, with single‐, two‐ and three‐portal techniques and some studies using fluoroscopic guidance. Due to inconsistent outcome reporting and limited stratified data, subgroup analysis was not feasible, and these techniques were pooled for comparison, potentially obscuring procedure‐specific differences. In addition, patients with more severe structural changes, such as extensive calcification or bony deformities, are more likely to undergo open surgery in clinical practice. This tendency may have introduced selection bias, as the open group could include more complex cases, limiting comparability between groups [11].

Furthermore, variability in surgical methods, rehabilitation protocols, patient age, comorbidities, and IAT severity likely contributed to additional heterogeneity. Post‐operative rehabilitation strategies were rarely standardized or clearly described, and were not incorporated into the surgical comparisons. This may have confounded outcomes such as return to activity and functional improvement. Future studies should aim to standardize rehabilitation protocols to minimize this potential source of bias.

To address these limitations, future research should prioritize high‐level studies, such as RCTs or prospective cohort studies, to generate more robust evidence for guiding surgical decision‐making in IAT management. Standardization of outcome measures and post‐operative care would further improve comparability and strengthen the reliability of future findings.

## CONCLUSIONS

This systematic review and meta‐analysis revealed no significant differences in long‐term functional outcomes between open and endoscopic surgery for IAT treatment. However, endoscopic surgery demonstrated clear advantages, including lower complication rates, reduced surgical failure, and a significantly shorter recovery time, particularly in return to daily activities and sports. These findings indicate that endoscopic surgery may be the preferable approach, particularly for high‐performance athletes and individuals requiring faster rehabilitation. Despite these promising results, the overall quality of evidence remains low, primarily due to the predominance of retrospective studies and methodological heterogeneity among the included studies. Additional high‐quality prospective cohort studies and RCTs, along with the development of optimized and standardized surgical and post‐operative protocols, are warranted to strengthen the evidence base for surgical decision‐making and to optimize treatment strategies for IAT.

## AUTHOR CONTRIBUTIONS

All authors contributed to the study conception and design. Material preparation, data collection and analysis were performed by Po‐Yuan Chen, I‐Shiang Tzeng and Chen‐Chie Wang. The first draft of the manuscript was written by Po‐Yuan Chen, Kai‐Chiang Yang and Chen‐Chie Wang, and all authors commented on previous versions of the manuscript. All authors read and approved the final manuscript.

## CONFLICT OF INTEREST STATEMENT

The authors declare no conflicts of interest.

## ETHICS STATEMENT

The ethics statement is not available.

## Supporting information


**Table S1.** PRISMA Checklist. **Table S2.** Keywords and search results in different databases. **Figure S1.** Funnel plot assessing publication bias for the primary outcome.

## Data Availability

All data generated or analyzed during this study are included in this published article.
